# A Deep Learning Approach for Distant Infrasound Signals Classification

**DOI:** 10.3390/s25072058

**Published:** 2025-03-26

**Authors:** Xiaofeng Tan, Xihai Li, Hongru Li, Xiaoniu Zeng, Tianyou Liu, Shengjie Luo

**Affiliations:** School of nuclear Engineering, Rocket Force University of Engneering, Xi’an 710025, China; tanxf2177@163.com (X.T.); lihr_huo@163.com (H.L.); xiaoniuzeng@163.com (X.Z.); tianyou_liu1@163.com (T.L.); losonjay@163.com (S.L.)

**Keywords:** infrasound signal classification, deep learning, noise reduction, station signal combination, CNN

## Abstract

Infrasound signal classification represents a critical challenge that demands immediate attention. Feature extraction stands as the core concept for enhancing classification accuracy in infrasound signal processing. However, existing feature extraction methodologies fail to meet the requirements for long-distance detection scenarios. To address these limitations, this study proposes a novel classification framework based on the spatiotemporal characteristics of infrasound signals. The proposed framework incorporates advanced signal processing techniques, signal enhancement algorithms, and deep learning architectures to achieve precise classification of infrasound signals. This paper designs three sets of comparative experiments, and the results demonstrate that the proposed method achieves a classification accuracy rate of 83.9% on chemical explosion and seismic infrasound datasets, outperforming eight other comparative classification methods. This substantiates the efficacy of the proposed approach.

## 1. Introduction

Infrasound, defined as low-frequency sound waves below 20 Hz, emanates from a diverse array of natural and anthropogenic sources and possesses the capability to traverse extensive distances within the natural environment [1]. Owing to its distinctive propagation attributes, infrasound is particularly well suited for remote event surveillance [2], regional and global natural disaster early warning, nuclear explosion monitoring, and earth science research. Infrasonic monitoring technology has emerged as one of the four pivotal technologies within the International Monitoring System (IMS) of the Comprehensive Nuclear-Test-Ban Treaty Organization (CTBTO) [3]. The primary interference sources in nuclear explosion infrasound detection predominantly consist of transient infrasound signals, including seismic infrasound and chemical explosion infrasound. These interfering signals exhibit remarkable similarities to nuclear explosion infrasound in multiple aspects, including temporal occurrence, propagation characteristics, waveform patterns, and spectral distribution. Consequently, the development of robust classification and identification methodologies for these interfering signals is crucial for enhancing the reliability of nuclear explosion monitoring. Empirical studies have demonstrated that infrasound signals generated by nuclear explosions are predominantly distributed within the frequency spectrum ranging from 0.01 Hz to 10 Hz [4], which constitutes the operational frequency range for most infrasound monitoring systems. In accordance with these findings, IMS has established a standardized sampling rate of 20 Hz [5] for its global infrasound monitoring network, ensuring the comprehensive acquisition and characterization of nuclear explosion-induced infrasound signatures.

The accuracy of classifying infrasound signals is fundamentally dependent on the extraction of effective features. Feature extraction methods for infrasound signals can be broadly categorized into single feature extraction and multi-valued feature extraction. Due to the inherent limitations of a single feature in capturing comprehensive information about infrasound events, the extraction of multi-valued features is more commonly adopted. Multi-valued features can be further subdivided into two distinct approaches. The first approach involves transforming the infrasound signal into a multi-valued feature with increased divisibility (e.g., power spectrum, spectral diagram, etc.) [6,7,8,9,10]. This transformation captures the feature evolution of the infrasound signal within a particular domain, thereby enhancing the feature’s expressiveness, albeit potentially at the cost of relevant information from other domains. The second approach entails extracting multiple single features and subsequently concatenating them to form a feature vector [11,12,13]. This technique enables the acquisition of features from various domains of infrasound signals. However, the selection of appropriate classification features requires prior domain knowledge and an efficient feature selection algorithm, thereby increasing the operational complexity.

In the domain of seismic signal classification, Zhao et al. [14] demonstrated how the efficacy of combining the three seismic components (P wave, SV wave, and SH wave) detected for the same event through deep learning methods can substantially enhance classification accuracy. This approach of integrating three components augments the redundant information inherent in seismic signal features. Although infrasound signals do not exhibit the same three-component structure as seismic signals, each infrasound array comprises multiple subarrays capable of simultaneously receiving multiple signals from the same event, which exhibit strong similarities. By amalgamating signals from the same station, it is feasible to increase the redundant information of infrasound signals. However, conventional deep learning networks cannot meet the requirements for infrasound signal classification. The Convolutional Block Attention Module (CBAM) [15] is a lightweight attention mechanism that effectively captures spatial and channel dimension information from infrasound signals. Long Short-Term Memory (LSTM), a specialized variant of Recurrent Neural Networks (RNNs), excels at extracting temporal-dependent features within infrasound signals [16]. By introducing both CBAM and LSTM modules into the infrasound signal classification network, this network can automatically acquire spatiotemporal information from infrasound signals simultaneously, thereby fully leveraging its advantages in processing infrasound signals.

In this study, we propose a novel classification framework for infrasound signals based on their spatiotemporal characteristics. Initially, we employ CEEMDAN and MSAM to eliminate the inherent low-frequency trend components and high-frequency noise in infrasound signals. Subsequently, we calculate the Welch power spectrum for each infrasound signal and combine two signals from the same event at the same station to create new infrasound samples. To enhance the infrasound data, the MVIDA algorithm is applied. Furthermore, we introduce CBAM and LSTM modules to design a deep learning network model tailored for infrasound signal classification. The proposed method achieves the integration of knowledge and deep learning at both data and feature levels, establishing a dual-driven framework that combines knowledge and data, with the aim of further enhancing the classification accuracy of infrasound signals.

## 2. Research Lines and Methodology

**Figure 1:**
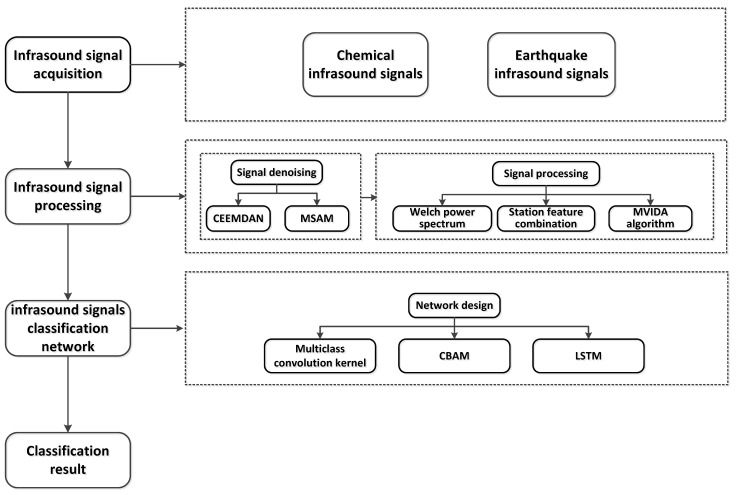
Technology roadmap.

Due to the complexity of infrasound signal propagation mechanisms and the multipath nature of their transmission, received signals exhibit significant classification challenges, and a universally applicable processing and classification framework has yet to be established. To address this issue, this study proposes a novel infrasound signal classification framework based on the spatiotemporal characteristics of infrasound signals. The framework incorporates advanced signal processing techniques, signal enhancement algorithms, and deep learning architectures to achieve efficient processing and accurate classification of infrasound signals. The proposed methodological framework is illustrated in Figure 1.

### 2.1. Infrasound Signal Acquisition

**Figure 2:**
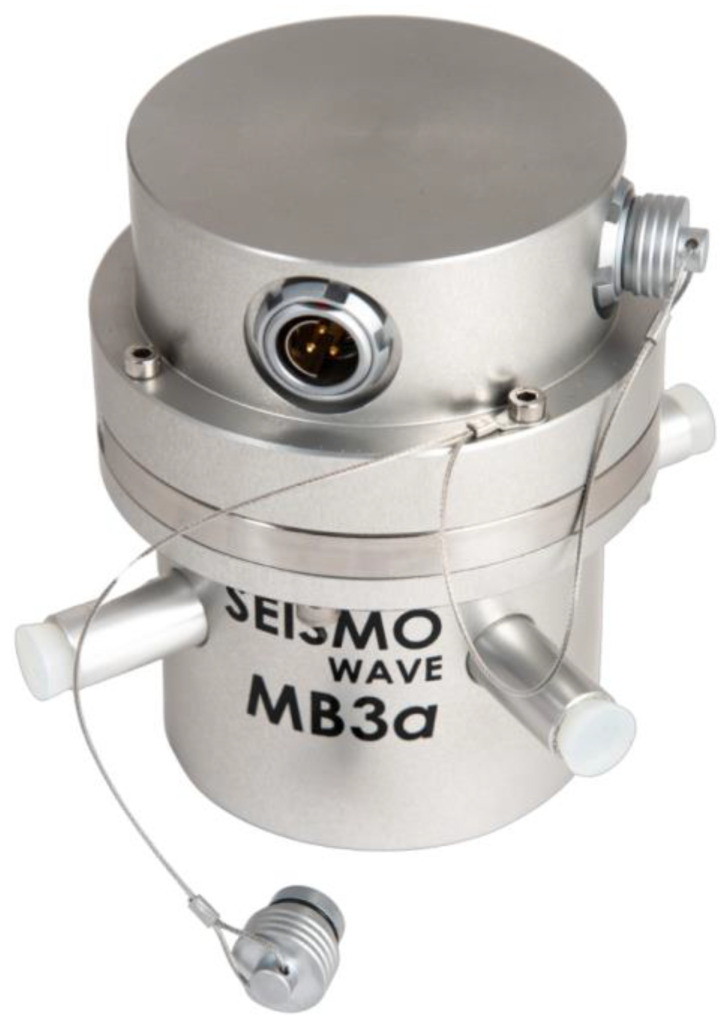
MB3a infrasound sensor.

This study utilizes the MB3a infrasound sensor, as illustrated in Figure 2, which is capable of effectively recording low-frequency acoustic signals within the range of 0.01 to 28 Hz, with a measurement sensitivity of 20 mV/Pa. The system operates at a sampling frequency of 20 Hz to ensure the accuracy and reliability of data acquisition.

We collected the infrasound data generated by chemical explosions and earthquakes, creating a catalog of waveforms that includes 36 events detected by stations, totaling 789 infrasound waveforms. This catalog comprises 386 samples from 28 chemical explosions and 403 samples from 8 seismic events. Each waveform in the catalog contains a complete event with a fixed length of 3000 data points corresponding to a 150 s time window.

Due to the limited number of infrasound observation signals utilized in this paper, we divided the infrasound data into three sub-data sets according to the event, ensuring that the infrasound data generated by the same event only exists in one subset. The purpose of this division is to minimize the correlation between test set data, training set data, and verification set data, thereby ensuring the reliability of network classification. The number of infrasound events and samples contained in the three sub-data sets are detailed in Table 1.

### 2.2. Infrasound Signal Preprocessing

#### 2.2.1. Signal Denoising

During propagation, infrasound signals are vulnerable to non-sound pressure fluctuations induced by turbulent interactions within the atmosphere, ground, or other obstacles [17]. In the transmission process, the infrasound signal will inevitably incorporate some noise and trend components, which can obscure important event information. Consequently, preprocessing the original infrasound signal to eliminate the influence of noise and trend items and enhance the signal-to-noise ratio (SNR) of the infrasound signal is essential to better accomplish the subsequent task of infrasound signal classification.

##### Complete Ensemble Empirical Mode Decomposition with Adaptive Noise

Infrasound events detected by a station are situated at a certain distance from that station. The propagation process subjects these signals to various random environmental noises, which can significantly impact their classification. Consequently, reducing noise from the original signals is a crucial aspect of data preprocessing. Given its robust performance in terms of self-adaptability and computational efficiency, the Complete Ensemble Empirical Mode Decomposition with Adaptive Noise (CEEMDAN) [18] is employed in this study for the decomposition of the original signal. CEEMDAN is a decomposition method proposed based on empirical mode decomposition (EMD) [19]. By integrating adaptive white noise into the EMD process multiple times, CEEMDAN effectively mitigates reconstruction errors and addresses potential mode aliasing issues inherent in EMD decomposition. The specific algorithmic steps can be referenced in the literature [20].

Subsequent to the decomposition process, the final residual can be represented as follows:

(1) $$r\left(t\right)=x\left(t\right)-\sum_{i=1}^{\lambda }{\bar{IMF}}_{i},$$

where $x\left(t\right)$ signifies the original infrasound signal, and $\bar{IMF_{i}}$ denotes Intrinsic Mode Function (IMF) component obtained after the derived from the i-th iteration of CEEMDAN decomposition.

##### The Mean of the Standardized Accumulated Modes

To effectively segregate the infrasound event signal from high-frequency noise and low-frequency trend terms, it is crucial to ascertain which component of the infrasound event information is more dominant. The conventional screening method involves calculating the similarity between each component and the original signal [21] or the energy ratio of each component [22]. These two methods are suitable for close detection of collected infrasound signals. Given that the original infrasound signal is more influenced by high-frequency noise and less by low-frequency trend terms, the correlation coefficient and energy ratio of the dominant component of infrasound event information to the original signal are notably higher than those of the dominant component of noise.

In light of this, this study opts for the mean of the standardized accumulated modes (MSAM) as a criterion for selecting IMF components, which can effectively screen out low-frequency trend items mixed in during the propagation process [23].

MSAM can be calculated as follows:

(2) $$h_{m}=\mathrm{mean}\left(\sum_{i=1}^{m}\left({\bar{IMF}}_{i}-\frac{\mathrm{mean}\left({\bar{IMF}}_{i}\right)}{\mathrm{std}\left({\bar{IMF}}_{i}\right)}\right)\right)\left(m\leq n\right),$$

where $\mathrm{mean}\left({\bar{IMF}}_{i}\right)$ is the mean value of ${\bar{IMF}}_{i}$, and $\mathrm{std}\left({\bar{IMF}}_{i}\right)$ is the standard deviation of ${\bar{IMF}}_{i}$. When the low-frequency trend term is not dominant among the current i IMF components, the $h_{m}$ value approaches zero. When the low-frequency trend term in the i+1 IMF component dominates, the $h_{m}$ value significantly deviates from zero. In this case, the higher-order components at this scale and above are regarded as low-frequency noise and trend terms. Removing these components can effectively eliminate their impact on infrasound signals.

##### Infrasound Signal Preprocessing Steps

**Figure 3:**
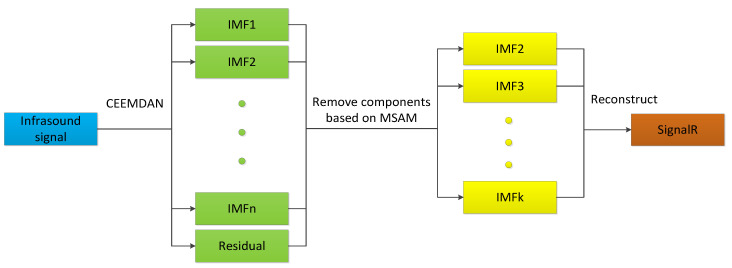
Noise reduction process diagram.

The process of signal noise reduction is illustrated in Figure 3, with the specific steps outlined as follows:

1. Signal decomposition: the CEEMDAN algorithm was employed to decompose the infrasound signal, yielding IMF components and residual components;
2. Noise identification: the MSAM was utilized to distinguish between noise and useful signal components. Components with MSAM values significantly deviating from zero were identified as low-frequency noise and trend terms, which were subsequently eliminated;
3. High-frequency noise removal: the IMF1 component, predominantly containing high-frequency noise, was discarded;
4. Signal reconstruction: the remaining IMF components were reconstructed to obtain the denoised signal.

**Figure 4:**
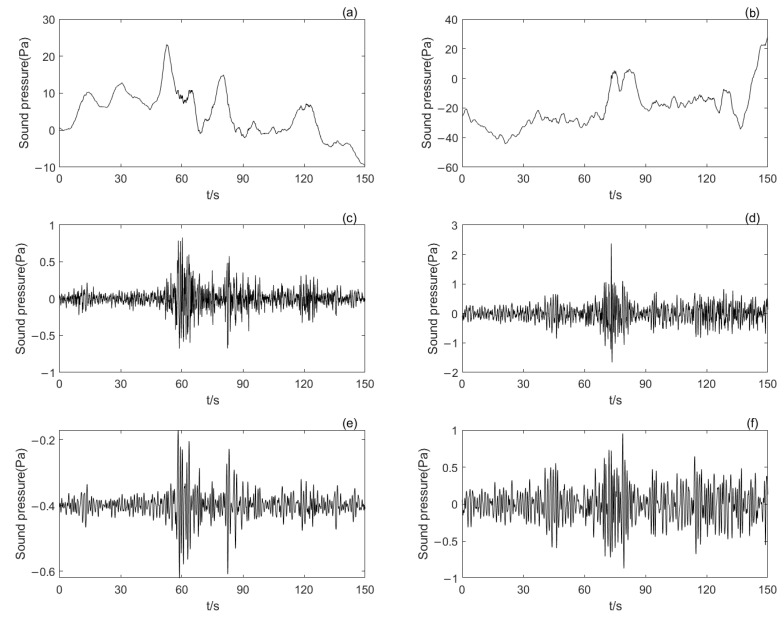
The figure shows the waveforms of the infrasound signal before and after preprocessing: (**a**) waveform of the chemical explosion infrasound signal; (**b**) waveform of the seismic infrasound signal; (**c**) waveform of the chemical explosion infrasound signal after removing low-frequency noise; (**d**) waveform of the seismic infrasound signal after removing low-frequency noise; (**e**) waveform of the chemical explosion infrasound signal after removing both low-frequency and high-frequency noise; (**f**) waveform of the seismic infrasound signal after removing both low-frequency and high-frequency noise.

Figure 4 illustrates the waveform variations of infrasound signals before and after noise reduction processing. As shown in Figure 4a,b, both chemical explosion and seismic infrasound signals contain distinct trend components that mask the critical information of infrasound events. In Figure 4c,d, after the removal of low-frequency noise, the infrasound event information becomes significantly more prominent, with its amplitude being one order of magnitude lower than that of the low-frequency background noise. This confirms the effectiveness of the CEEMDAN–MSAM method in eliminating low-frequency background noise. Figure 4e,f present the reconstructed signals after removing the IMF1 component, indicating that IMF1 does not contain high-frequency noise. Removing this component can significantly enhance the clarity of infrasound event information.

The proposed method in this study effectively reduces high-frequency noise and low-frequency trend components in infrasound signals while retaining the essential characteristics of the events, thereby facilitating clearer observation and analysis of potential infrasound event information.

#### 2.2.2. Signal Processing

To address the challenge of accurate infrasound signal classification and mitigate the limitations of data-driven approaches, this study introduces a knowledge-driven strategy into the signal processing procedure to enhance classification accuracy. The knowledge-deep neural network fusion is implemented at two levels: (1) at the data level through the application of the MVIDA algorithm for infrasound data enhancement, and (2) at the feature level via the computation of Welch power spectral density and station signal combinations.

##### Welch Power Spectrum

The power spectrum, as a crucial method for analyzing acoustic signals, elucidates the relationship between signal power and frequency. When used as a feature, the power spectrum can effectively highlight the differences between seismic infrasound signals and chemical explosion infrasound signals. The Welch method [24], an improvement on Bartlett’s method, is effective in spectral estimation. By segmenting the data and increasing the window overlap, this method effectively reduces the variance of spectral estimation and avoids serious signal distortion [25]. Let the length of the infrasound signal $x\left(n\right)$ be N, divided into L segments, each containing M points.

The power spectrum of each segment of Welch method can be expressed as follows [26]:

(3) $$P_{PER}^{i}\left(\omega \right)=\frac{1}{MU}{\left|\sum_{n=0}^{M-1}x^{i}\left(n\right)d\left(n\right)e^{-j\omega n}\right|}^{2}$$

where n is the number of sampling points of the infrasound signal, and U is the normalization factor to obtain the asymptotic unbiased estimator of the Welch power spectrum. U is specifically expressed as follows:

(4) $$U=\frac{1}{M}\sum_{n=0}^{M-1}d^{2}\left(n\right)$$

$d\left(n\right)$ is a window function, so the average power spectrum can be expressed as follows:

(5) $$P_{PER}\left(f\right)=\frac{1}{MUL}\sum_{i=1}^{L}{\left|\sum_{n=0}^{M-1}x^{i}\left(n\right)d\left(n\right)e^{-j2\pi fn}\right|}^{2}$$

The window function employed was Hanning, with a window length of 512 points and a sample overlap of 256 points. The FFT length was also set to 512 points. The length of the infrasound signal is 3000 sampling points. Following feature extraction, the power spectrum length for each infrasound signal was determined to be 257 points.

##### Station Signal Combination

The acquisition of infrasound event signals is predominantly facilitated through sensor elements within an infrasound array, with each array typically comprising four to ten individual elements. To ensure waveform consistency during signal correlation processing and to mitigate data transmission complexity, it is standard practice to limit the triangular matrix scales to a maximum of one wavelength and the multi-lattice scales to generally not exceed half a wavelength; otherwise, folding effects may occur [27]. Consequently, there is a substantial correlation between infrasound signals detected by different elements within the same station array. Furthermore, it is probable that infrasound generated by identical events will be simultaneously received by stations positioned at various locations. Although the infrasound signals obtained by different stations may exhibit significant variations due to the influence of disparate sensors and noise during the propagation process, the potential for underlying connections between these signals remains uncertain.

This pertinent information is frequently employed in infrasound event detection tasks to suppress irrelevant background fluctuations, identify infrasound events, and characterize infrasound sources. However, in the context of infrasound event classification, this relevant information is typically overlooked, with each element’s detected signal being treated as an independent infrasound sample. Although this approach has a minimal impact on the infrasound classification task, the judicious utilization of this relevant information could also enhance the task’s efficacy.

**Figure 5:**
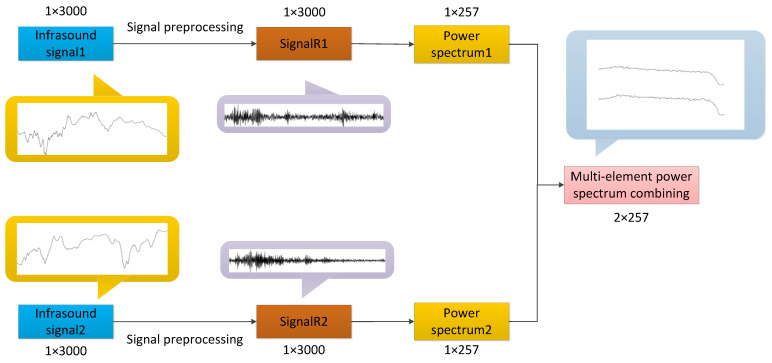
Schematic representation of the signal combination process at the same station.

Figure 5 illustrates the combined processing workflow for infrasound signals. Initially, two infrasound signals detected by different elements of the same array are preprocessed using the noise reduction method presented in this paper. The results indicate that the processed waveforms are similar but exhibit a time lag. This discrepancy arises because both infrasound signals correspond to the same event, while the detecting elements are spatially separated. Subsequently, the power spectra of each signal are independently extracted, revealing high similarity between them. Finally, the two power spectra are combined and fed into the network as inputs from different channels. This approach not only increases redundant information but also enhances the distinctiveness of individual sample features, thereby improving the performance of feature classification.

##### Mixed Virtual Infrasound Data Augmentation

The acquisition of infrasound event signals relies on infrasound sources. Due to the low frequency of the events, obtaining a large number of signals is challenging. Therefore, data augmentation methods are necessary to expand the dataset. In this study, we primarily utilized the Mixed Virtual Infrasound Data Augmentation (MVIDA) algorithm [28] to augment our dataset. The MVIDA algorithm computes a new virtual infrasonic signal based on the signal from the same station array. This virtual signal can be approximately regarded as representing the power spectrum received by a new array element in that staton array.

Following mathematical derivation, the power spectral density $P_{i+1}\left(\omega \right)$ corresponding to the i+1-th array element $x_{i+1}\left(n\right)$ can be formulated as follows:

(6) $$P_{i+1}\left(\omega \right)=P_{S}\left(\omega \right)+P_{NO_{i+1}}$$

where $P_{S}\left(\omega \right)$ represents the power spectral density of the observed infrasonic signal $s\left(n\right)$, and $P_{NO_{i+1}}$ denotes the noise power spectral density in the vicinity of the $x_{i+1}\left(n\right)$ array element.

$k\left(2\leq k<l\right)$ elements are randomly selected from the same station, and the power spectral density of infrasonic signals received by each array element is linearly combined to obtain a new virtual power spectral density $P_{y}\left(\omega \right)$, the algorithm formula can be expressed as follows:

(7) $$P_{y}\left(\omega \right)=\frac{\sum_{i=1}^{k}\alpha_{i+1}P_{i+1}\left(\omega \right)}{\sum_{i=1}^{k}\alpha_{i+1}}$$

The mathematical expression for the resulting virtual array power spectral density $P_{y}\left(\omega \right)$ can be formulated as follows:

(8) $$P_{y}\left(\omega \right)=P_{S}\left(\omega \right)+P_{NO_{i+1}}^{\prime }$$

The parameter $P_{NO_{i+1}}^{\prime }$ can be interpreted as the weighted average of the noise power spectral density among different array elements. This characteristic indicates that the virtual power spectrum $P_{y}\left(\omega \right)$ generated by MVIDA demonstrates significant similarity to the real power spectrum $P_{i+1}\left(\omega \right)$. The virtual spectrum $P_{y}\left(\omega \right)$ can effectively approximate the power spectral density of infrasound signals received by newly configured array elements within the station. This algorithm exhibits two notable advantages: (1) it maintains clear physical interpretability, and (2) it enhances the reliability of data processing.

#### 2.2.3. Construction of Infrasound Signal Classification Data Set

**Figure 6:**
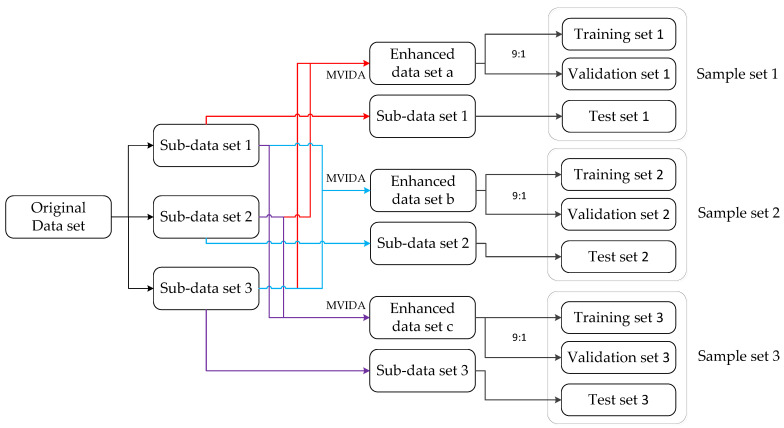
Schematic diagram of dataset division steps.

As shown in Figure 6, the MVIDA algorithm is implemented to enhance the infrasound signal dataset and construct the classification dataset. The methodological workflow comprises the following sequential steps:

1. Generate training set 1 and verification set 1 based on subsets 2 and 3’s infrasound samples. That is, sub-data sets 1 and 2 are merged into sample set A. Then after expanding sample set A about fifty times using MVIDA algorithm, we obtain sample set 1. This sample set was then randomly shuffled and divided into training set 1 and verification set 1 at a ratio of approximately nine to one;
2. Place infrasound samples from sub-data sets into test-set without enhanced virtual samples;
3. The process of generating training set 2 and verification set 2 is repeated based on infrasonic samples from sub-data sets 1 and 3, while test set 2 is generated based on infrasonic samples from sub-data set 2. Similarly, training set 3 and verification set 3 are generated using infrasound samples from sub-data sets 1 and 2, with test set 3 being generated based on infrasonic samples from sub-data set 3. This results in three sets of training, verification, and test data. The classification results for each sample set are calculated separately to obtain the final average classification results. The number of infrasonic samples in each data set before and after enhancement can be found in Table 2.

To ensure the reliability of network classification, the dataset was partitioned according to event categories. This event-based partitioning strategy minimizes potential correlations between training, validation, and test sets, effectively addressing the inherent similarities among infrasound signals originating from the same event.

### 2.3. Classification Model Based on Neural Network

**Figure 7:**
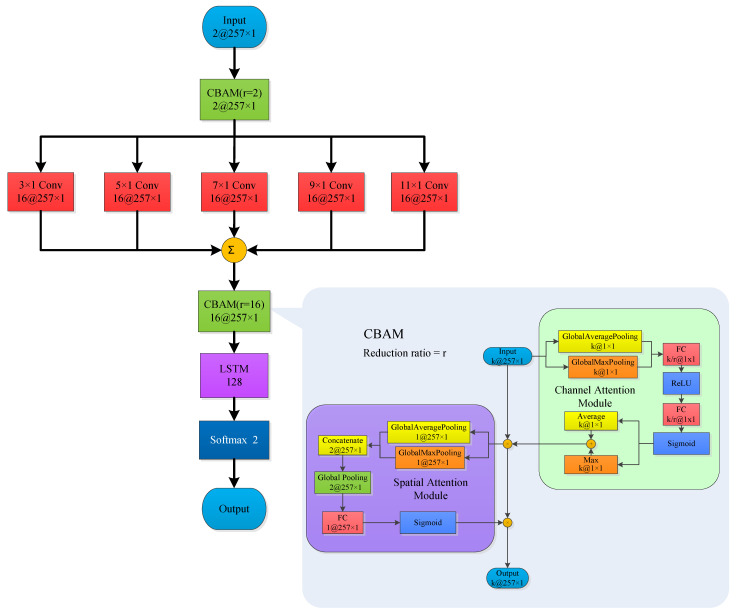
Structure of PCMLN.

Based on the spatiotemporal characteristics of infrasound signals, this study proposes a Parallel Convolutional Kernel, CBAM, and LSTM Network (PCMLN) for infrasound signal classification. The comprehensive network architecture is presented in Figure 7.

This study designs a multi-level network architecture: the first layer consists of a Convolutional Block Attention Module (CBAM) with a reduction ratio of two, which implements preliminary feature extraction of the input data. The second layer comprises five parallel convolutional layers with convolution kernels of sizes 3×1, 5×1, 7×1, 9×1, and 11×1, respectively, facilitating multi-scale feature extraction. The outputs from these parallel convolutional layers are then aggregated to integrate information across different scales. Let the output of the parallel convolutional layers be denoted as $O_{i}$, where $i\in \left[1,2,3,4,5\right]$. The aggregated output $O_{n}$ can be represented as follows:

(9) $$O_{n}=\sum_{i=1}^{5}O_{i}$$

Following the parameter fusion layer, a third CBAM layer with a reduction ratio of 16 is designed to achieve advanced feature selection. To enhance the time series feature extraction capabilities, a single-layer LSTM module with 128 nodes is incorporated into the network, employing the tanh activation function. Finally, a softmax classification layer is designed to achieve accurate discrimination between chemical explosions and seismic events.

The primary objective of the initial CBAM is to acquire the characteristics of signal combination weighted in both the channel domain and spatial domain. To achieve this, five parallel convolution layers are incorporated to merge convolution features of different scales into new features, which have been found to be more effective than a single feature [29]. Employing CBAM once more can enhance the scale feature channels and spatial weights that are beneficial for classification tasks, while suppressing those that are not conducive to such tasks. This operation not only improves the network’s classification capability but also enhances its robustness in selecting the scale of the convolutional kernel. Additionally, LSTM is introduced to ensure that the network pays attention to both temporal and spatial characteristics, leveraging their respective strengths in processing infrasonic signals and ultimately improving classification accuracy.

## 3. Results and Discussions

### 3.1. Experimental Procedure

In this study, we implement the infrasound signal classification task using Python 3.9 and TensorFlow 2.5 frameworks. The experimental procedure consists of the following methodological steps:

1. Preprocess infrasound signals: Firstly, CEEMDAN is applied to decompose the sub-acoustic signal, yielding a set of IMF components and a residual component. Secondly, MSAM is used to distinguish between noise and useful signal components. If the MSAM value significantly deviates from zero, the higher-order IMF components above this threshold are identified as low-frequency noise and trend terms and are removed. Additionally, the IMF1 component, which is predominantly dominated by high-frequency noise, is also eliminated. Subsequently, the remaining IMF components are reconstructed to obtain the denoised signal. Finally, a Welch power spectrum of the reconstructed signal is calculated;
2. Combine infrasound signals: A new sample of infrasound signal is obtained by combining pairs according to the station configuration;
3. Data set division: The infrasound sample is divided into three sub-data sets, ensuring that the infrasound data generated by the same event only exists in one subset. Each time, the training set and validation set are obtained based on two of the sub-data sets and enhanced by the MVIDA algorithm, while the test set is generated from the remaining sub-data set. This process is repeated three times to obtain three different sets of training, validation, and test sets;
4. Model training and refinement: The PCMLN model is trained using the training set, and then amended based on the validation set;
5. Classification using trained network model: The trained network model is used to classify the test set. This process is repeated three times, and then average classification results are calculated from these three repetitions.

### 3.2. Evaluation Parameters of Model Performance

Four statistics—true positives, false positives, false negatives, and true negatives—can be obtained using a confusion matrix, as shown in Table 3.

The accuracy (*ACC*), F1 values, sensitivity (*TPR*), and specificity (*TNR*) are commonly used to evaluate the performance of classification models. In this paper, the four statistics of *TP*, *FP*, *TN*, and *FN* were used to calculate these indicators to evaluate the classification model, specifically defined as follows:

(10) $$ACC=\frac{TP+TN}{TP+FP+TN+FN}$$

(11) $$R=\frac{TP}{TP+FN}$$

(12) $$P=\frac{TP}{TP+FP}$$

(13) $$F1=\frac{2\times R\times P}{R+P}$$

(14) $$TPR=\frac{TP}{TP+FN}$$

(15) $$TNR=\frac{TN}{TN+FP}$$

### 3.3. Analysis of Experimental Results

We conducted three sets of experiments to verify each processing step’s effectiveness in this study. Firstly, we designed an ablation experiment for infrasound signal processing to validate both noise reduction and feature combination operations’ effectiveness. Secondly, an ablation experiment of neural networks was designed to verify CBAM’s and LSTM’s impact on network model performance. Finally, we compared our trained network model with five classical lightweight CNNs as well as three CNNs applied to infrasonic classification in order to evaluate our proposed neural network model’s performance.

#### 3.3.1. Infrasound Signal Processing Ablation Experiment

To validate the effectiveness of infrasound signal processing operations (noise reduction and feature combination), we established three experimental groups for comparison. The first group served as the baseline, where no noise reduction was performed, but signal feature combination was conducted. The second group underwent noise reduction but did not perform signal feature combination, while the third group completed both noise reduction and signal feature combination. For each experimental group, we sequentially extracted the Welch power spectrum of the infrasound signal samples as input features for the network, applied the MVIDA algorithm for data augmentation, and utilized an improved CNN model [30] for training and classification. This network is a one-dimensional convolutional neural network specifically developed by our team in 2021 for classifying infrasound signals.

**Figure 8:**
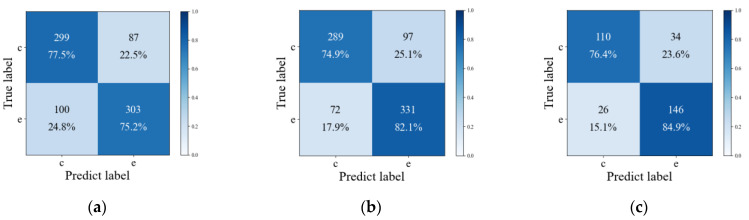
Confusion matrices of classification results for three groups of operations (c for chemical explosion, e for earthquake). (**a**) Confusion matrix of classification results without noise reduction and feature combination processing. (**b**) Confusion matrix of classification results with noise reduction but without feature combination processing. (**c**) Confusion matrix of classification results with both noise reduction and feature combination processing.

The confusion matrix for each set of experiments is illustrated in Figure 8 Each row represents the actual label (c for chemical explosion, e for earthquake), while each column represents the label predicted by the network model. The diagonal entries represent correct classifications (e.g., upper left corner indicates the number of correctly labeled chemical explosion infrasound signals and their proportion among all such signals), whereas off-diagonal entries indicate misclassifications (e.g., lower left corner shows the number of seismic infrasound signals incorrectly labeled as chemical explosion infrasound signals and their proportion among all seismic infrasound signals).

In order to provide a more detailed evaluation of model performance, three evaluation indices—f-score, sensitivity (*TPR*), and specificity (*TNR*)—were added based on *ACC*. The f-score represents the harmonic average of precision (*P*) and recall (*R*). Sensitivity and specificity reflect the classification accuracy of chemical explosion and seismic infrasound signals, respectively. Table 4 was designed to display the indicators of the three sets of operation experiments. The classification accuracy were 76.3%, 78.6%, and 81.0%, respectively. It was observed that compared with data sets without preprocessing and combination operations, both the *ACC* and f-score were effectively improved after only preprocessing operations. Additionally, from the perspective of sensitivity and specificity, while there was a slight decrease in classification accuracy for chemical explosion infrasound signals, there was a significant improvement in classification accuracy for seismic infrasound signals after preprocessing operations. This indicates that the pretreatment operation may sacrifice a small part of divisibility for chemical explosion infrasound signals (1.1–2.6%), but it is an effective operation overall as it significantly increases divisibility for seismic infrasonic signals (6.9–8.3%). After completing combined operations, all four indexes showed improvement, which proves their effectiveness. Overall, datasets that completed both operations showed a 4.7% increase in *ACC* and a 2.3% increase in f-score compared to baseline results, indicating that both preprocessing and signal combination can help improve the classification accuracy of infrasound signals.

#### 3.3.2. Ablation Experiment of Neural Network Model

In addition to the comparative experiments on infrasound signal processing operations, we conducted ablation studies on the neural network model to validate the classification performance of the parallel multi-convolutional kernel module, CBAM module, and LSTM module. The ablation study consists of three configurations: (1) the first configuration excludes the LSTM module from the classification model, (2) the second configuration removes the CBAM module, and (3) the third configuration employs the complete PCMLN as the classification model. The experimental results from the fourth configuration of the infrasound signal processing comparison experiment serve as our baseline. For all four groups, we completed two types of operations: preprocessing and signal combination. We then extracted the Welch power spectrum features from each group’s infrasound signal samples for use as input features in our network. Additionally, we employed the MVIDA algorithm for data enhancement. The specific classification results are presented in Table 5.

Compared to PCMLN, the four indexes of the classification model show a significant decrease after using the CBAM module and LSTM module alone. This indicates that the information lost during feature extraction in both types of networks—spatial information or time-dependent information—significantly impacts their classification performance. However, it is observed that the classification accuracy of the LSTM module decreases more significantly than that of the CBAM module. This difference may be attributed to spatial information having a stronger impact on separability than time-dependent information when completing the task of classifying infrasonic signals. Therefore, it can be considered more important to focus on improving spatial information extraction when undertaking infrasound signal classification tasks.

Compared to the baseline network, the PCMLN network model designed in this paper has shown improvements in all four evaluation indexes (ranging from 1.1% to 4.9%), demonstrating the effectiveness of the network for infrasound signal classification. The experimental results indicate that combining the CBAM and LSTM modules leads to higher classification accuracy compared to using a single type of network. This further validates the model’s ability to focus on both spatial and temporal features, ultimately enhancing classification accuracy.

#### 3.3.3. Model Performance Comparison Experiment

In order to further validate the classification performance and reliability of the PCMLN model, we selected five classical lightweight convolutional neural networks and three CNNs used for infrasound classification as comparators. These include LeNet-5, AlexNet, VGG16, GoogLeNet, ResNet18, improved CNN, improved LeNet-5, and improved AlexNet [31]. The convolution layers of classical lightweight CNNs are all within 18 layers, and CNNs suitable for infrasound classification are all improvements made on the LeNet-5 and AlexNet networks, respectively. Additionally, in order to conduct the training and testing of the data in this study, we converted all two-dimensional convolution kernels to one-dimensional ones.

**Figure 9:**
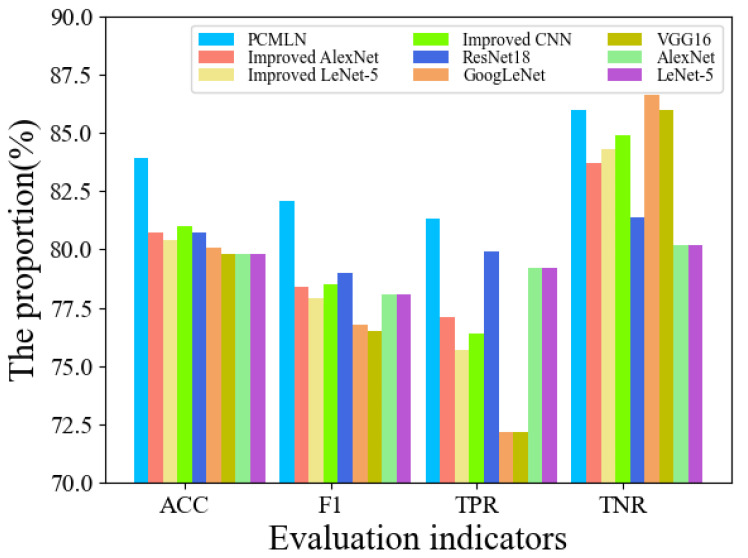
Comparison of classification results of two types of infrasound events by different classification models.

Table 6 and Figure 9 present the classification results of nine different classification models for the test sets of chemical explosion and seismic infrasound signals. The PCMLN model achieved a classification accuracy of 83.9% and an F1 value of 82.1% for the test set, which are both the highest among the nine models and significantly superior to others. This demonstrates that the model has the best classification performance for both seismic infrasonic signals and chemical explosion infrasonic signals. Additionally, it ranked first in TPR and second in TNR, indicating its overall stability.

We can further observe from Table 6 that the classification accuracy of classical CNN for infrasound signals is relatively consistent, hovering around 80%, with the F1 value also ranging between 76.5% and 79.0%. It is evident that increasing the number of convolutional layers has minimal impact on the classification effectiveness for infrasound signals with limited samples. Therefore, it is more reasonable to opt for a simple CNN network directly or make enhancements based on this foundation. Leng et al. and Tan et al. made improvements to LeNet-5, while Yuan enhanced AlexNet. The results indicate that compared to the previous models, the improvements are not significant and may even show a decrease in performance. This could be attributed to differences in datasets used as well as potential impacts on the classification performance of transitioning from a two-dimensional network to a one-dimensional network.

In general, for the task of classifying infrasound signals with small samples, increasing the complexity of CNN does not necessarily improve classification accuracy but may lead to overfitting problems. The PCMLN used in this paper consists of a total of seven convolutional layers, and none of the aforementioned networks used for infrasound signal classification has more than eight convolutional layers. Therefore, we suggest that the number of convolutional layers in the network should be limited to eight when designing a CNN for infrasound classification tasks with small samples.

**Figure 10:**
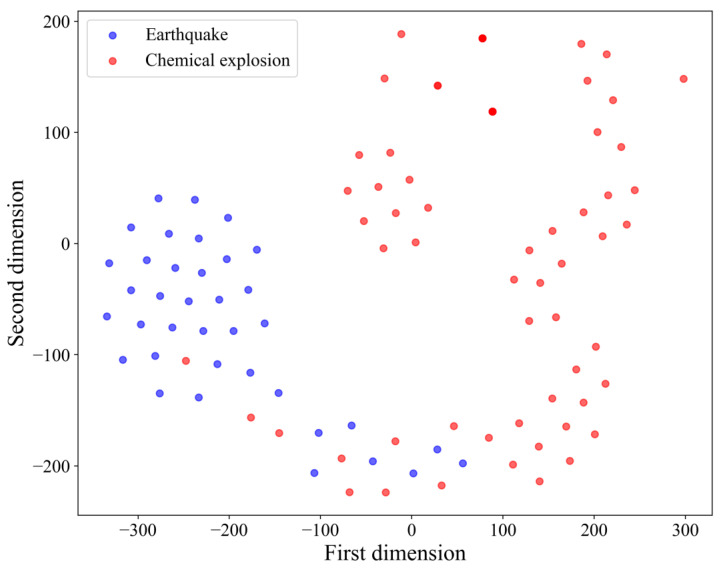
T-SNE dimensionality reduction visualization of the classification results.

To elucidate the misclassification of infrasound samples, we applied t-distributed Stochastic Neighbor Embedding (t-SNE) to visualize the output of the classification network by reducing its dimensionality, as shown in Figure 10. The results indicate that there is a certain degree of overlap between the seismic infrasound features and the chemical explosion infrasound features extracted by the PCMLN network. Further analysis reveals that the seismic infrasound features exhibit a highly convergent distribution pattern in the low-dimensional space, characterized by small intra-class distances. In contrast, the chemical explosion infrasound features display a relatively divergent distribution with larger intra-class distances. This difference is likely due to the fact that chemical explosion data encompass multiple subcategories (e.g., missile explosions, ammunition depot explosions, and fireworks explosions). The power spectrum characteristics of infrasound signals generated by these different subcategories vary, leading to a more dispersed feature distribution. In contrast, seismic infrasound signals typically have a more consistent generation mechanism and propagation characteristics, resulting in a more concentrated feature distribution that is more readily recognized by classification models.

## 4. Conclusions

Based on the spatiotemporal characteristics of infrasound signals, this study proposes a novel infrasound signal classification method. The proposed method achieves the integration of knowledge and deep learning at both data and feature levels, establishing a dual-driven framework that combines knowledge and data. Experimental results demonstrate its superior classification performance when applied to the classification task of chemical explosions and seismic infrasound signals.

We used our own collection of chemical explosion and earthquake infrasound signals as a classification dataset, which includes nearly 800 samples. Based on the characteristics of the infrasound station combination and data enhancement techniques, we divided sub-data sets for classification and evaluation using cross-validation. We designed three groups of experiments to verify the effectiveness of each step in this paper. The experimental results demonstrate that both pre-processing and signal combination operations can help improve the classification accuracy of infrasound signals. The PCMLN model designed in this paper can pay attention to both spatial and temporal characteristics at the same time, effectively identifying separability between infrasounds from chemical explosions and earthquakes with a classification accuracy of 83.9%.

In this study, we employed a dataset comprising 789 samples of chemical explosion and earthquake infrasound signals collected by our research group. Based on the characteristics of the infrasound station configurations and data augmentation techniques, we utilized cross-validation to partition the dataset into sub-data sets for classification and evaluation. To validate the effectiveness of each step in our proposed methodology, we designed three experimental groups. The results demonstrate that both preprocessing and signal combination operations significantly enhance the classification accuracy of infrasound signals. The PCMLN model developed in this study integrates both spatial and temporal features, effectively identifying the separability between infrasounds from chemical explosions and earthquakes. The classification accuracy achieved was 83.9%, indicating that the proposed framework exhibits strong generalization capability for distinguishing between these two types of infrasound signals. However, the cross-scenario generalization ability of our model for other types of events (e.g., bolide explosions, mine blasts) requires further validation. Future work will focus on expanding the dataset to include a broader range of event types and exploring cross-domain transfer learning or domain adaptation methods to improve the model’s adaptability and robustness across different scenarios.

This study utilizes a one-dimensional feature extraction method and a neural network architecture suitable for processing one-dimensional data. However, considering that the neural network demonstrates superior performance in two-dimensional feature processing, the current one-dimensional network approach may limit the model’s performance optimization to some extent. Based on this, our subsequent research will focus on exploring methods to transform one-dimensional infrasound signals into two-dimensional feature maps. Through technical approaches such as time–frequency analysis or feature reconstruction, we aim to construct two-dimensional attribute maps that incorporate temporal and spatial characteristics. This improvement is expected to significantly enhance the network’s capability to extract spatiotemporal features, thereby improving the model’s classification accuracy and generalization performance.

## Figures and Tables

**Table 1:** Each sub-data set includes information on infrasound events, the number of samples, and the total.

	Chemical Explosion		Earthquake	
	Number of Events	Number of Samples	Number of Events	Number of Samples
Sub-data set 1	10	123	4	121
Sub-data set 2	12	144	2	154
Sub-data set 3	6	119	2	128

**Table 2:** Comparison of the quantity of infrasound data before and after enhancement in each sample set.

		Chemical Explosion		Earthquake	
		Sample Size Before Augmentation	Sample Size After Augmentation	Sample Size Before Augmentation	Sample Size After Augmentation
Sample set 1	Training set	86	4947	106	6299
	Validation set	10	549	12	699
	Test set (nonenhancement)	48	-	54	-
Sample set 2	Training set	82	4456	92	5672
	Validation set	9	495	10	630
	Test set (nonenhancement)	53	-	70	-
Sample set 3	Training set	91	4465	111	5260
	Validation set	10	496	13	584
	Test set (nonenhancement)	43	-	48	-

**Table 3:** Confusion matrix list.

Actual\Forecast	Positive	Negative
Positive	True Positive (*TP*)	False Negative (*FN*)
Negative	False Positive (*FP*)	True Negative (*TN*)

**Table 4:** Results of infrasound signal processing ablation experimental.

Operations		ACC	f-Score	TPR	TNR
Noise Reduction	Feature Combination				
×	×	76.3%	76.2%	77.5%	75.2%
√	×	78.6%	77.4%	74.9%	82.1%
√	√ ^1^	**81.0%**	**78.5%**	**76.4%**	**84.9%**

^1^ × indicates that the operation was not used, while √ indicates that the operation was used.

**Table 5:** Results of ablation experiment with neural network model.

Network Module		ACC	f-Score	TPR	TNR
CBAM	LSTM				
√	×	82.3%	80.4%	79.9%	84.3%
×	√	69.6%	65.5%	63.2%	75.0%
√	√	83.9%	82.1%	81.3%	86.0%
× (baseline)	× ^1^ (baseline)	81.0%	78.5%	76.4%	84.9%

^1^ × indicates the absence of the module in the network, while √ indicates the presence of the module in the network.

**Table 6:** Classification results of two types of infrasound events by different classification models.

	ACC	f-Score	TPR	TNR
Based on classic CNN				
LeNet-5	80.7%	78.3%	76.4%	84.3%
AlexNet	79.8%	78.1%	79.2%	80.2%
VGG16	79.8%	76.5%	72.2%	86.0%
GoogLeNet	80.1%	76.8%	72.2%	86.6%
ResNet18	80.7%	79.0%	79.9%	81.4%
Classification CNN based on infrasound				
Improved CNN	81.0%	78.5%	76.4%	84.9%
Improved LeNet-5	80.4%	77.9%	75.7%	84.3%
Improved AlexNet	80.7%	78.4%	77.1%	83.7%
PCMLN	83.9%	82.1%	81.3%	86.0%

## Data Availability

Data related to this study are available and can be obtained by contacting the corresponding author.
